# Comparison of two techniques for measuring *Demodex folliculorum* and *Demodex brevis* in rosacea patients: standardized skin surface biopsy vs. direct microscopic examination

**DOI:** 10.1017/S0031182025100632

**Published:** 2025-09

**Authors:** Jaime Pérez Wilson, Sebastián Andreani Figueroa, Soledad Aspillaga Vergara, Juana Benedetto Eblen, Cristóbal Lecaros Cornejo, Viviana García Ramos, Diego Méndez Villanueva, Daniel Velásquez Muñoz, Paulina Ríos, Angelo Di Gennaro, Tomás Olivares, Jorge Olivares

**Affiliations:** 1 Faculty of Medicine, Clínica Alemana-Universidad del Desarrollo, Vitacura, Santiago, Chile; 2 Dermatology Department, Medicien, Las Condes, Santiago, Chile; 3 Faculty of Medicine, Pontificia Universidad Católica de Chile, Santiago, Chile; 4 Dermatology Laboratory, Clínica Alemana, Vitacura, Santiago, Chile

**Keywords:** *Demodex* spp, direct microscopic examination, rosacea, standardized skin surface biopsy

## Abstract

Rosacea is a chronic inflammatory skin disease affecting approximately 5.4% of the world population. Among its pathogenic factors is infestation by *Demodex* spp. Standardized skin surface biopsy (SSSB) and direct microscopic examination (DME) are widely used methods to measure *Demodex* spp density (Dd); however, there is no agreement on the method of choice, nor the prevalence of infestation in rosacea patients. This study compared both techniques in rosacea patients. A prospective study was conducted with 61 patients diagnosed with rosacea by dermatologists from two dermatology centres. Dd was evaluated using SSSB and DME in each patient. Results, median sampling time and reported pain were analyzed using appropriate statistical methods. The median Dd was significantly higher with SSSB (11 mites/cm^2^) compared to DME (1 mites/cm^2^; *P* < 0.001). Infestation (>5 mites/cm^2^) was detected in 64% of patients with SSSB and in 28% with DME (*P* < 0.001). The median sampling time was longer for SSSB (60 s) than for DME (30 s; *P* < 0.001). Both methods were associated with mild pain, slightly lower with DME (*P* = 0.033). SSSB proved more effective than DME for detecting *Demodex* spp. in rosacea, identifying a greater total number of mites and a higher percentage of infestation. Up to 64% of rosacea patients showed infestation with *Demodex* spp. using the SSSB technique. The results reinforce the use of SSSB as the standard technique for diagnosing *Demodex* spp. infestation in rosacea patients.

## Graphical abstract

**Figure:**
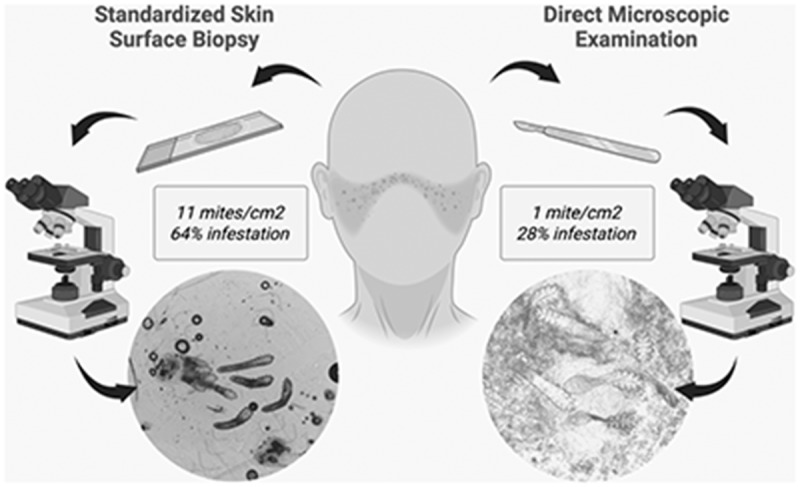

## Introduction

Rosacea is a chronic inflammatory disease characterized by persistent facial erythema, papules, pustules, telangiectasia and flushing. It affects approximately 5.4% of the world population (Gether et al., 2018). While its pathogenesis is not fully understood, a higher prevalence of *Demodex* spp. infestation has been associated with rosacea, particularly in erythematotelangiectatic and papulopustular subtypes (Chang and Huang, 2017). Literature reports are variable regarding the percentage of rosacea patients with *Demodex* spp. infestation, ranging from 30% to 100% (Perrigouard et al., 2013; Lee et al., 2016; Ghanadan et al., 2023).

The commensal mites *Demodex folliculorum* and *Demodex brevis* are considered part of the normal skin microbiota, residing exclusively in human pilosebaceous follicles with a prevalence of up to 100% (Forton and Seys, 1993; Wei et al., 2024). Demodicosis is defined as high density of these mites on the skin, associated with compatible clinical characteristics.

Various measurement methods exist, being Standardized skin surface biopsy (SSSB) the most widely used found in literature. Other methods include direct microscopic examination (DME), reflectance confocal microscopy (RCM), fluorescence advanced videodermoscopy (FAV), adhesive tape method, polymerase chain reaction (PCR) and biopsy (Pérez-Wilson et al., 2021).

Infestation is typically defined as a *Demodex* density (Dd) of >5 mites per cm^2^, measured using SSSB or DME interchangeably (Aşkın and Seçkin, 2010; Bunyaratavej et al., 2016; Yun et al., 2017). However, there is no consensus on the most appropriate method for measurement.

This study aimed to compare the SSSB and DME techniques in 61 patients diagnosed with rosacea and determine the percentage of *Demodex* spp. infestation in patients with this condition.

## Materials and methods

A prospective comparative study was conducted following approval from the Ethics Committee of Clínica Alemana de Santiago.

This observational study was designed and reported in accordance with the Strengthening the Reporting of Observational Studies in Epidemiology (STROBE) guidelines to ensure transparency and integrity in the presentation of results. The sample size of 61 patients was determined based on previous studies evaluating Dd in rosacea patients, ensuring adequate statistical power for comparative analysis.

Sixty-one adult patients with a clinical diagnosis of rosacea, confirmed by dermatologists, who consulted from August 2022 to May 2023 at the Dermatology Department of Clínica Alemana de Santiago and Medicien Dermatology Centre, Santiago, Chile, were proposed for enrolment.

Only patients over 18 years old with erythematotelangiectatic and papulopustular rosacea with compatible dermoscopy were included.

Patients with ocular or phymatous rosacea, other dermatological conditions, or recent treatment (<3 months) for *Demodex* spp. were excluded.

Written informed consent was obtained from all patients. All 61 patients underwent both techniques on the facial region.

### Detection methods

**Figure 1.**
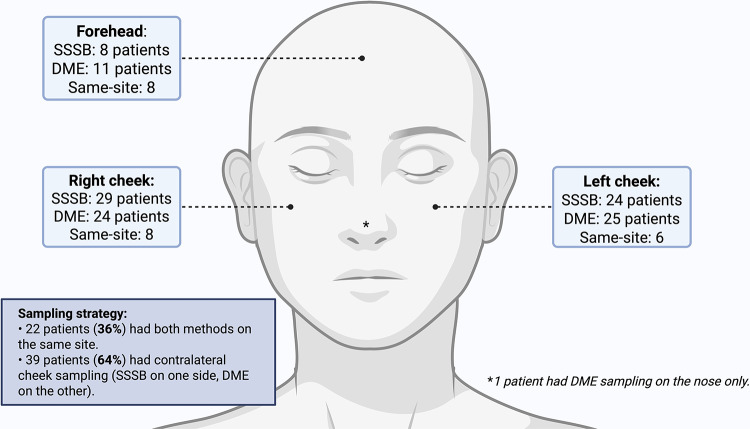
Anatomical distribution and sampling strategy of standardized SSSB and DME in rosacea patients. Sampling sites were selected based on dermoscopic findings suggestive of *Demodex* infestation. The figure illustrates the number of patients per technique and anatomical location, as well as whether sampling was performed on the same or contralateral sites.

Sampling sites were selected based on dermoscopic findings, prioritizing areas with visible lesions and features suggestive of *Demodex* infestation, such as *Demodex* tails, follicular plugs and perifollicular scaling (*Lallas et al., 2014*). Both SSSB and DME were performed in each patient to assess Dd. In 22 patients (36%), both techniques were applied to the same anatomical site (forehead, right cheek or left cheek). In the remaining 39 patients (64%), sampling was performed on contralateral cheeks (Figure 1).

For SSSB, a standard 1 cm^2^ area was marked on a glass slide. A drop of cyanoacrylate adhesive was applied to the opposite side of the slide and held against the skin for 1 min. After allowing the adhesive to dry, the slide was removed and one or two drops of immersion oil were applied. It was then covered with a coverslip.

For DME, a 1 cm^2^ area of affected skin with compatible dermoscopy was scraped using a scalpel. The sample obtained was transferred to a drop of Chlorazol black E and covered with a coverslip.

Samples obtained by both methods were examined under optical microscopy at 10×, 20× and 40× magnification.

### Data collection

Along with the results and the technique used, data on age, sampling site, sampling time and pain were recorded. Pain intensity was measured for each technique using a visual analog scale (VAS) from 0 to 10.

### Statistical analysis

Statistical analysis was performed on the Dd, sampling time and pain reported by patients. The Shapiro–Wilk test was used to assess normality. For non-normally distributed, paired data, the Wilcoxon signed-rank test was applied to evaluate statistical significance.

Additionally, a subgroup comparison of the magnitude of difference in mite counts between SSSB and DME stratified by sampling site (same-site vs. contralateral-site) was performed using the Mann–Whitney U-test.

Interquartile range (IQR) was reported as 25th–75th percentiles. A *P*-value < 0.05 was considered statistically significant.

## Results

Results were obtained from a sample of 61 rosacea patients diagnosed by dermatologists from the two participating centres, of whom 36 were women and 25 men. Baseline characteristics are summarized in Table 1.

**Table 1.** Baseline characteristics of patients

Characteristic	Gender		
	Female *N* = 36	Male *N* = 25	Total *N* = 61
Age^a^	39 (31.43)	45 (38.55)	40 (32.47)
*Demodex* spp. density by technique^a^			
SSSB	8 (0.15)	26 (2.69)	11 (1.35)
DME	1 (0.4)	2 (1.7)	1 (0.7)
Pain by technique^a^			
SSSB	2 (1.3)	3 (1.4)	2 (1.4)
DME	1 (1.2)	1 (1.3)	1 (1.3)
Sampling site for SSSB^b^			
Cheek	31 (86%)	22 (88%)	53 (87%)
Nose	–	–	–
Forehead	5 (14%)	3 (12%)	8 (13%)
Sampling SITE for DME^b^			
Cheek	29 (81%)	20 (80%)	49 (80%)
Nose	0 (0%)	1 (4.0%)	1 (1.6%)
Forehead	7 (19%)	4 (16%)	11 (18%)

a Median (Interquartile range); years; pain intensity measured using Visual Analog Scale.

b *N* (%).

Abbreviations: DME, Direct Microscopic Examination; SSSB, Standardized Skin Surface Biopsy.

All 61 eligible patients who met the inclusion criteria were enrolled in the study, with no exclusions. There was no loss of participants during the study, and all completed both the SSSB and DME procedures.

### Mite density

**Figure 2.**
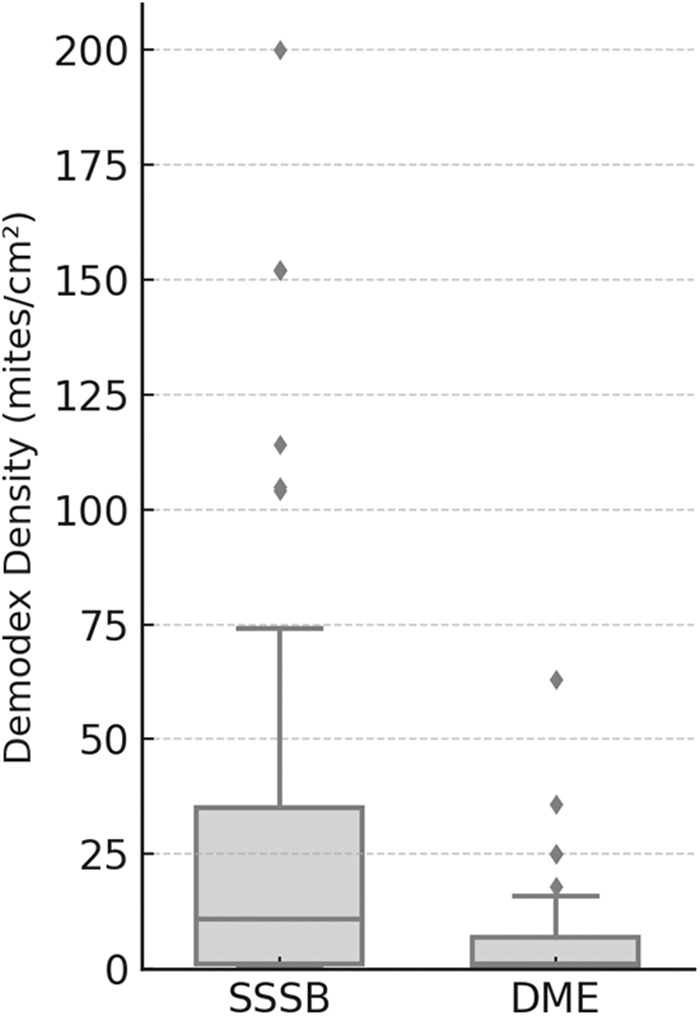
Comparison of *Demodex* spp. Density measured by SSSB and DME in rosacea patients. Boxplots show median values, interquartile ranges and outliers for each technique.

Statistical analysis revealed a median Dd of 11 mites/cm^2^ (IQR: 1–35) with SSSB and 1 mite/cm^2^ (IQR: 0–7) with DME, with a significant difference between methods (p < 0.001) (Figure 2).

This difference was consistent between patients sampled at the same anatomical site and those with contralateral-site sampling, with no statistically significant variation between groups (*P* = 0.081).

### Demodex spp. Infestation

**Figure 3.**
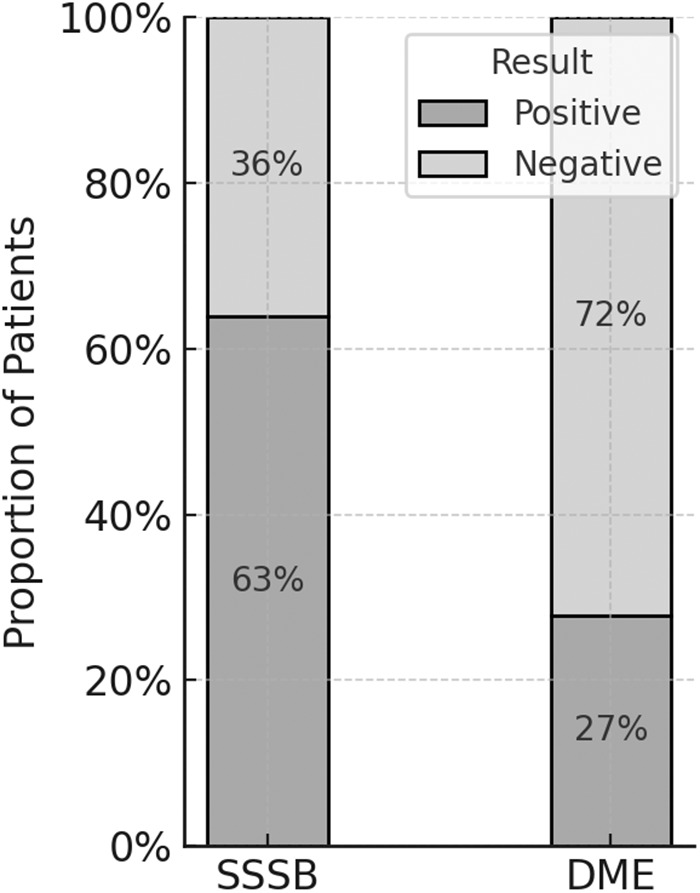
Proportion of rosacea patients classified as positive or negative for *Demodex* spp. Infestation according to the diagnostic technique used: SSSB and DME. Bar segments indicate relative frequencies of positive and negative cases per method.

*Demodex* spp. infestation (defined as a density > 5 mites/cm^2^) was detected in 64% of patients using SSSB (*n* = 39) and in 28% using DME (*n* = 17), with statistically significant differences (*P* < 0.001) (Figure 3).

### Median sampling time

**Figure 4.**
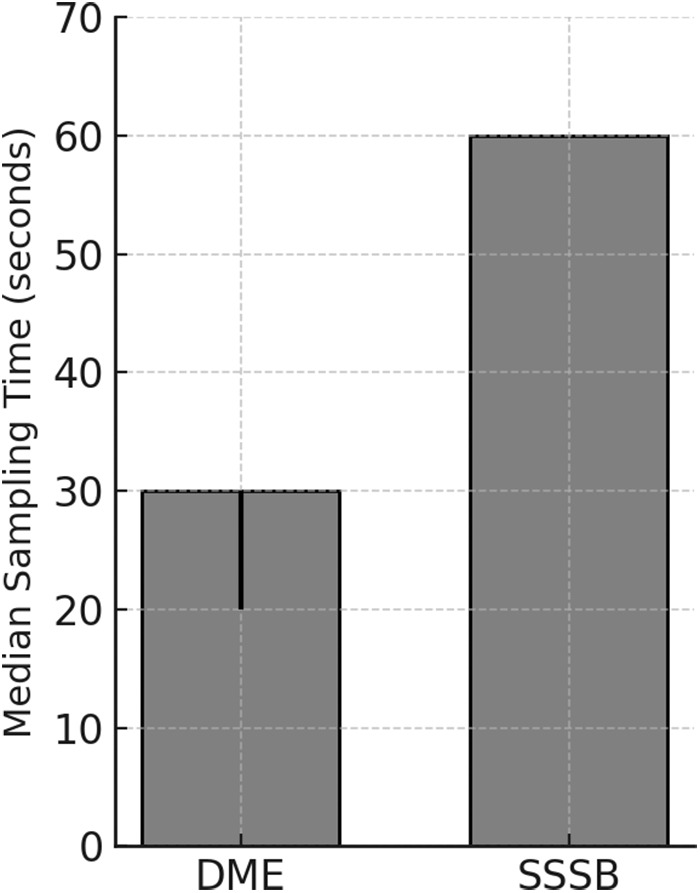
Median sampling time in seconds for DME and SSSB. Bars represent the typical duration required to perform each technique.

The sampling time for SSSB was standardized at 60 s for all patients, in accordance with the requirements of the technique. For DME, the median sampling time was 30 s (IQR: 20–30), which was significantly shorter (*P* < 0.0001) (Figure 4).

### Reported pain

**Figure 5.**
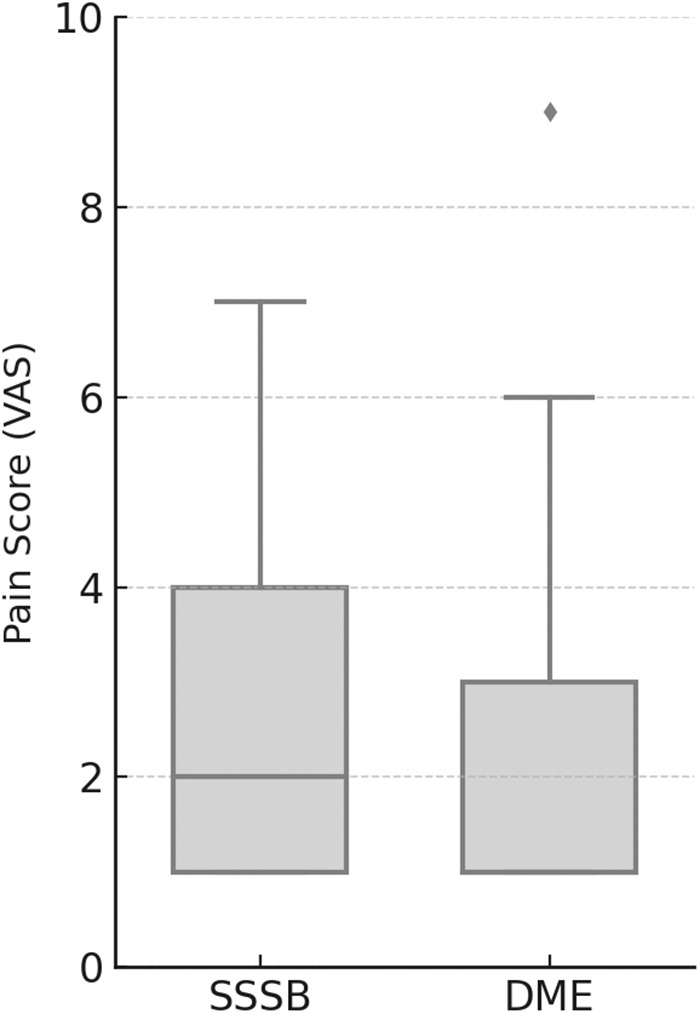
Pain scores reported by patients undergoing SSSB and DME. Boxplots represent median values, interquartile ranges, and outliers based on a visual analog scale (VAS) from 0 to 10.

Reported pain (VAS): The median pain reported was 2.0 (IQR: 1–4) for SSSB and 1.0 (IQR: 1–3) for DME, with a statistically significant difference between techniques (*P* = 0.0064). Both values fall below 3 points, indicating mild pain for both procedures (Figure 5).

## Discussion

Numerous methods are available to evaluate Dd, with SSSB and DME being the most commonly employed in clinical practice due to their simplicity and cost-effectiveness (Pérez-Wilson et al., 2021). This study directly compared both techniques in rosacea patients using a standardized, dermoscopy-guided protocol, revealing significant differences in diagnostic yield.

SSSB demonstrated a significantly higher positivity rate (64% vs. 28%) and median mite density (11 [IQR 1–35] vs. 1 [IQR 0–7] mites/cm^2^) compared to DME. These findings confirm the superior sensitivity of SSSB for detecting *Demodex* mites and are consistent with existing literature supporting its diagnostic utility in rosacea (Aşkın and Seçkin, 2010; Forton and Maertelaer, 2017).

To date, only 3 studies have directly compared SSSB and DME (Aşkın and Seçkin, 2010; Bunyaratavej et al., 2016; Yun et al., 2017). Aşkın and Seçkin found SSSB to be more effective, Bunyaratavej observed no statistically significant difference, and Yun reported higher sensitivity with DME. These discrepancies likely reflect methodological differences, such as sampling site selection, dermoscopic guidance, slide preparation and analysis, rather than true diagnostic equivalence. Our study, which employed a standardized and dermoscopy-guided protocol, contributes additional evidence supporting the diagnostic advantage of SSSB in rosacea patients.

**Figure 6.**
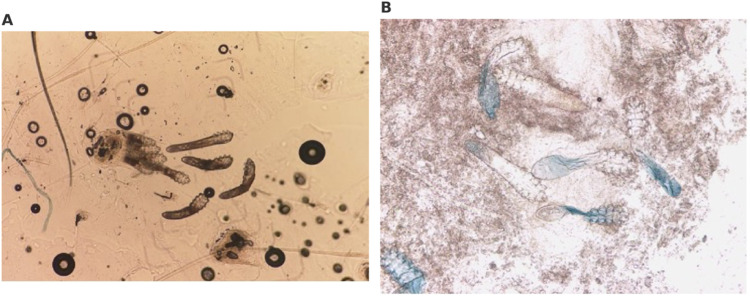
Microscopic appearance of *Demodex* spp. Under different sampling techniques. (A) SSSB showing multiple intact demodex mites extracted with follicular contents. (B) DME revealing fewer mites, often fragmented and surrounded by background debris.

Microscopic examination further supported this difference in performance. SSSB, through the adhesive action of cyanoacrylate, extracts follicular contents with minimal fragmentation, allowing for clearer identification of intact mites. In fact, previous studies have shown that even greater detection rates can be achieved by performing two consecutive SSSB samples on the same site (Forton and Maertelaer, 2017). In contrast, DME frequently produces fragmented structures and is more affected by keratin and background debris, complicating visualization (Figure 6). Although DME may theoretically access deeper layers of the skin, including sebaceous glands where *Demodex brevis* resides (Yun et al., 2017), its overall sensitivity remains limited.

Non-invasive diagnostic tools, such as RCM, allow direct visualization of mites in vivo and have shown promising sensitivity in both diagnosis and treatment monitoring (Turgut Erdemir et al., 2014; Sattler et al., 2015). Similarly, molecular detection methods like PCR have demonstrated higher sensitivity than conventional microscopy, including in low-density cases (Casas et al., 2012; Trave et al., 2024). However, PCR does not provide accurate quantification of mite density, as it detects DNA rather than intact mites. These techniques are not yet widely available and may involve higher costs, limiting their routine use in clinical practice.

Biological factors may also account for variability in Dd. A prospective study by Özdemir Çetinkaya et al. (2024) showed higher mite counts in patients with erythematotelangiectatic rosacea than in those with papulopustular disease. Other host-related factors such as age, immune status and sebum production have also been implicated in mite proliferation (Chang and Huang, 2017).

Although SSSB took longer to perform than DME, both procedures were completed in under 1 min and associated with only mild discomfort. The slight difference in reported pain and execution time is unlikely to impact clinical decision-making, particularly when balanced against diagnostic sensitivity.

This study excluded phymatous and ocular rosacea and did not compare Dd across subtypes, which may limit the generalizability of the results. Additionally, while SSSB sampling time was standardized at 60 s, this did not include preparation steps such as marking the site or allowing the adhesive to dry, so the actual procedure time may have been slightly underestimated compared to DME.

In view of these findings, we support the use of SSSB as the preferred method for quantifying Dd in rosacea patients. Its superior detection capacity, reproducibility and compatibility with clinical workflow make it a practical standard. Future studies should aim to refine diagnostic protocols and investigate integrated approaches with RCM or PCR.

## Conclusions

This study concludes that SSSB is a more effective and accurate technique than DME for detecting *Demodex* spp. in rosacea patients, with a median mite density nearly 10 times higher. DME consistently underestimates Dd compared to SSSB.

Demonstrating up to 64% infestation by *Demodex* spp. in the studied patients reinforces the association between *Demodex* spp. and rosacea, highlighting the importance of selecting an appropriate diagnostic method for its implications in management and treatment.

These findings led to the consideration of SSSB as the standard technique in the participating centres.
